# Cardiovascular health in pediatric heart transplant patients

**DOI:** 10.1186/s12872-022-02575-z

**Published:** 2022-04-01

**Authors:** Carmel Bogle, Amanda Marma Perak, Sarah J. Wilkens, Alaa Aljiffry, Karen Rychlik, John M. Costello, Donald M. Lloyd-Jones, Elfriede Pahl

**Affiliations:** 1 Ann and Robert H. Lurie Children’s Hospital of Chicago, Chicago, IL USA; 2 Northwestern University Feinberg School of Medicine, Chicago, IL USA; 3 University of Louisville School of Medicine, Louisville, KY USA; 4 Children’s Healthcare of Atlanta, Atlanta, GA USA; 5 Medical University of South Carolina Children’s Health, Charleston, SC USA; 6 University of Maryland Children’s Heart Program, Baltimore, MD USA

**Keywords:** Cardiometabolic, Pediatric, Heart transplant, Cardiovascular health

## Abstract

**Background:**

Ideal “cardiovascular health” (CVH)–optimal diet, exercise, nonsmoking, BMI, BP, lipids, and glucose—is associated with healthy longevity in adults. Pediatric heart transplant (HT) patients may be at risk for suboptimal CVH.

**Methods:**

Single-center retrospective study of HT patients 2003–2014 who survived 1 year post-transplant. Five CVH metrics were collected at listing, 1, 3 and 5 years post-transplant (diet and exercise were unavailable). CVH was scored by summing individual metrics: ideal = 2, intermediate = 1, and poor = 0 points; total scores of 8–10 points were considered high (favorable). CVH was compared between HT patients and the US pediatric population (GP) utilizing NHANES 2007–2016. Logistic regression was performed to examine the association of CVH 1 year post-transplant with a composite adverse outcome (death, re-listing, coronary vasculopathy, or chronic kidney disease) 3 years post-transplant.

**Results:**

We included 110 HT patients (median age at HT: 6 years [range 0.1–21]) and 19,081 NHANES participants. CVH scores among HT patients were generally high at listing (75%), 1 (74%), 3 (87%) and 5 (76%) years post-transplant and similar to GP, but some metrics (e.g., glucose) were worse among HT patients. Among HT patients, CVH was poorer with older age and non-Caucasian race/ethnicity. Per 1-point higher CVH score, the demographic-adjusted OR for adverse outcomes was 0.95 (95% CI, 0.7–1.4).

**Conclusions:**

HT patients had generally favorable CVH, but some metrics were unfavorable and CVH varied by age and race/ethnicity. No significant association was detected between CVH and adverse outcomes in this small sample, but study in a larger sample is warranted.

**Supplementary Information:**

The online version contains supplementary material available at 10.1186/s12872-022-02575-z.

## Introduction

### Background

Survival after pediatric heart transplant (HT) has improved over time, with more than half of recipients now living at least 17 years after HT [1, 2]. Two of the main factors limiting long-term survival among pediatric HT recipients are coronary allograft vasculopathy (CAV) [3] and chronic kidney disease (CKD) [1]. Evidence-based preventive strategies are elusive, but observational data in pediatric and adult HT recipients suggest that traditional cardiovascular risk factors, such as dyslipidemia and obesity, are associated with increased risk for these complications [4–6]. Previous studies have shown that pediatric HT recipients have elevated levels of total cholesterol (TC) and triglycerides and lower levels of high density lipoprotein (HDL-C) when compared to the general pediatric population [7–10]. As dyslipidemia is associated with CAV development in adult HT recipients, afflicted patients may be at increased cardiovascular risk [4, 5, 11–14]. CKD, also known to increase mortality and morbidity in both adult and pediatric HT recipients, is also exacerbated by cardiovascular risk factors [15, 16]. Despite the continued trend of increased obesity seen in the general pediatric population, with 17% of children between the ages of 2–19 years classified as obese and 5.8% as morbidly obese [17], there have been limited data over the past decade examining multiple cardiovascular risk factors and their implications for HT outcomes in pediatric HT recipients [5–7, 9, 13, 14, 18].

In 2010, the American Heart Association (AHA) defined “ideal cardiovascular health (CVH)” as optimal levels of seven metrics (Life’s Simple 7): diet, physical activity, nonsmoking, body mass index (BMI), blood pressure (BP), cholesterol, and glucose [19]. In the general population, higher levels of CVH, quantified by a CVH “score,” have been associated with greater longevity and health-related quality of life and lower healthcare costs [20–22]. However, no study has examined CVH or its association with outcomes in the pediatric HT population.

Therefore, we sought to (1) describe the distribution of CVH among pediatric HT recipients, (2) compare these CVH levels with those in the general US pediatric population, and (3) examine the associations of CVH with a composite adverse outcome including CKD, CAV, re-listing for HT or death.

## Methods

### Study design, setting and participants

We conducted a retrospective single-center study at a large-volume urban pediatric HT center in Chicago, Illinois. Patients who received a primary orthotopic HT from January 1, 2003 to December 31st, 2014 were eligible for the study. January 1. 2003 was selected as the start date to minimize era effects from the widespread change in immunosuppression from cyclosporine to tacrolimus-based therapy after 2000 at our center, as well as routine addition of induction prophylaxis at that time [31]. Patients were excluded from the analyses if they died within the first year after HT, or transferred care out of the institution for > 18 months after HT.

For the comparison population, publicly available, de-identified data from the National Health and Nutrition Examination Survey (NHANES) were used. Data from survey cycles 2007–2008 through 2015–2016 were used to match the era of 3- to 5-year follow-up in the HT group (2006–2017), and ages 0–18 years were included to mirror the age distribution of the HT patients. This comparison group is termed GP (general population).

The Lurie Children’s Hospital Institutional Review Board approved the study protocol and the need for written informed consent was waived.

### Variables: cardiovascular health metrics and cardiovascular health score

CVH was assessed via medical record abstraction at multiple time points among HT recipients: time of listing, 1, 3, and 5 years post-HT. For the comparison population, as NHANES is a cross-sectional study, CVH assessment was at a single time point. Of the seven AHA metrics used to define CVH, diet and physical activity were not consistently available in the electronic medical record for the HT recipient population are therefore not included in the CVH score calculation for either group (HT or GP), as has been done in prior studies using clinical datasets [23, 24]. CVH for both the HT sample and GP were defined using the remaining five metrics: smoking status (self-reported), BMI, BP, TC, and fasting blood glucose. In NHANES, CVH metric availability varied by age group as follows: BMI available in all participants, TC available for ages ≥ 6 years, BP available for ages ≥ 8 years, smoking status available for ages ≥ 12 years, and fasting glucose available in a subset of participants ≥ 12 years old. In addition to the five CVH metrics, lipid fractions including low density lipoprotein cholesterol (LDL-C), high density lipoprotein cholesterol (HDL-C), and triglycerides (TG) among both HT recipients and the GP were secondarily assessed, as these may be relevant for CAV pathogenesis [5, 7, 8, 14].

Individual CVH metrics were each categorized as ideal, intermediate, or poor using AHA’s pediatric definitions (Table 1). A composite CVH score was calculated by assigning points to each metric, with 0 points for poor levels, 1 point for intermediate levels, and 2 points for ideal levels, for a total score of 0–10 points (Table 1). Composite CVH scores were then categorized as: 0–4 points = low, 5–7 points = moderate, and 8–10 points = high (favorable), as previously reported [25]. HT recipients that were placed on a statin for hyperlipidemia or a blood pressure medication had one point subtracted from their respective CVH metric score, as recommended by the AHA CVH definition for “treated” metrics.

**Table 1:** Definitions of cardiovascular health

	Ideal	Intermediate	Poor
*Individual cardiovascular health metric categories*			
Smoking	Never smoked	–	Ever smoked
Body mass index	< 85th percentile	85–95th percentile	> 95th percentile
Blood pressure	< 90th percentile	90–95th percentile^a^	> 95th percentile^b^
Total cholesterol	< 170 mg/dl	170–199 mg/dl^c^	> 199 mg/dl^d^
Fasting blood glucose	< 100 mg/dl	100–125 mg/dl^e^	> 125 mg/dl^f^

	High	Moderate	Low
*Total cardiovascular health score categories*			
	8–10	5–7	0–4

^a^Includes patients with < 90th percentile blood pressure while on blood pressure medication

^b^Includes patients with ≥ 90th percentile blood pressure while on blood pressure medication

^c^Includes patients with total cholesterol < 170 mg/dl on statins

^d^Includes patients with total cholesterol 170–199 mg/dl on statins

^e^Includes patients with fasting blood glucose < 100 mg/dL while on diabetes mellitus medication

^f^Includes patients with fasting blood glucose ≥ 100 mg/dL while on diabetes mellitus medication

*CVH* cardiovascular health

### Covariates

Demographic variables collected included age, sex, and race/ethnicity. For HT recipients, medical history variables were also collected from the medical record, including original cardiac diagnosis, listing date, transplant date, presence and grade of CAV, blood urea nitrogen, creatinine, medications, and diabetes diagnosis.

### Adverse outcomes among HT recipients

Adverse outcomes including CAV, CKD, re-listing and death, were abstracted from patient medical records through 12/31/17. CAV grade was obtained from annual clinical visit notes and cardiac catheterization reports. CAV grading utilized the International Society of Heart and Lung Transplantation (ISHLT) guidelines with modifications from a recent Pediatric Heart Transplant Society (PHTS) study, in which CAV ranges from CAV Grade 0 (not significant) to CAV Grade 3 (severe) [11]. CKD, was defined as present if the estimated glomerular filtration rate (eGFR) was < 60 mL/min/1.73 m^2^ during routine outpatient visits [16, 26, 27].

### Statistical Methods

To characterize CVH among HT recipients, CVH metrics and CVH scores were calculated and categorized (Table 1). CVH score was counted as a continuous variable, which has been done in multiple large-scale studies [28–30]. Continuous variables were reported as mean ± standard deviation, and categorical variables were reported as counts (percent). CVH was described for the overall group of HT recipients and also for subgroups defined a priori by age at transplant (0–1, 2–5, 6–7, 8–11, and ≥ 12 years), race/ethnicity, and cardiac diagnosis (congenital heart disease or cardiomyopathy). Tests for differences in CVH among subgroups were considered exploratory, given small subgroup sizes.

To compare CVH between HT recipients and the GP, NHANES survey weights (e.g., fasting weights for glucose analyses) were used to generate accurate population estimates. CVH metrics were compared quantitatively and CVH composite scores (low, moderate, high) were compared between groups qualitatively; due to limitations of statistical inference for comparisons between weighted (NHANES) and un-weighted (HT recipients) data from different populations, statistical significance of differences were not tested.

Finally, to test for an association between CVH score and adverse outcomes among HT recipients, univariate and multivariable logistic regression was performed with the exposure CVH at year 1 post-HT and the outcome adverse HT at year 3 post-HT (year 3 was chosen to maximize available sample size). The composite adverse HT outcome included CKD, CAV, re-listing or death. Participants were censored if a non-fatal adverse outcome occurred prior to 1-year post-HT. Both crude and adjusted (for sex, age, transplant age, race/ethnicity and primary cardiac diagnosis) odds ratio for the adverse outcome at year 3 post-HT were calculated, per 1 point higher (more favorable) CVH score at 1 year post-HT.

Statistical analyses were performed using SAS version 9.4 (SAS Institute, Cary, NC). Two-tailed P values < 0.05 were considered statistically significant.

## Results

### Study participants

A total of 127 patients received a primary HT from January 1st 2003 to December 31^st^ 2014. Seventeen patients were excluded (10 transferred post-HT care to another center for > 18 months; 7 died prior to 1-year post-HT); thus 110 patients were included in the analytic sample. The median age at transplant was 6 years (range 0.1–21 years), 44% were female, 43% were Non-Hispanic Caucasian and 42% had a primary diagnosis of congenital heart disease. The GP comparison sample included 19,081 children (Table 2).

**Table 2:** Study participant characteristics

	Heart transplant recipients, N (%)	NHANES sample, N (%)
Total sample size	110	19,081
Sex		
Male	62 (56)	9,763 (51)
Female	48 (44)	9,318 (49)
Ethnicity/race		
Non-Hispanic Caucasian	63 (57)	5,495 (29)
Non-Hispanic African American	20 (18)	4,497 (23)
Hispanic	26 (24)	6,808 (36)
Other/unknown	1 (1)	2,281 (12)
Age, years		
0–1	39 (35)	3,600 (19)
2–5	16 (15)	4,335 (23)
6–11	24 (22)	6,267 (33)
12+	31 (28)	4,879 (25)
Cardiac history		
Cardiomyopathy	64 (58)	N/A
Congenital heart disease	46 (42)	N/A

NHANES, National Health and Nutrition Examination Survey, 2007–2016. See text (Methods) for details

### CVH among HT recipients

**Fig. 1:**
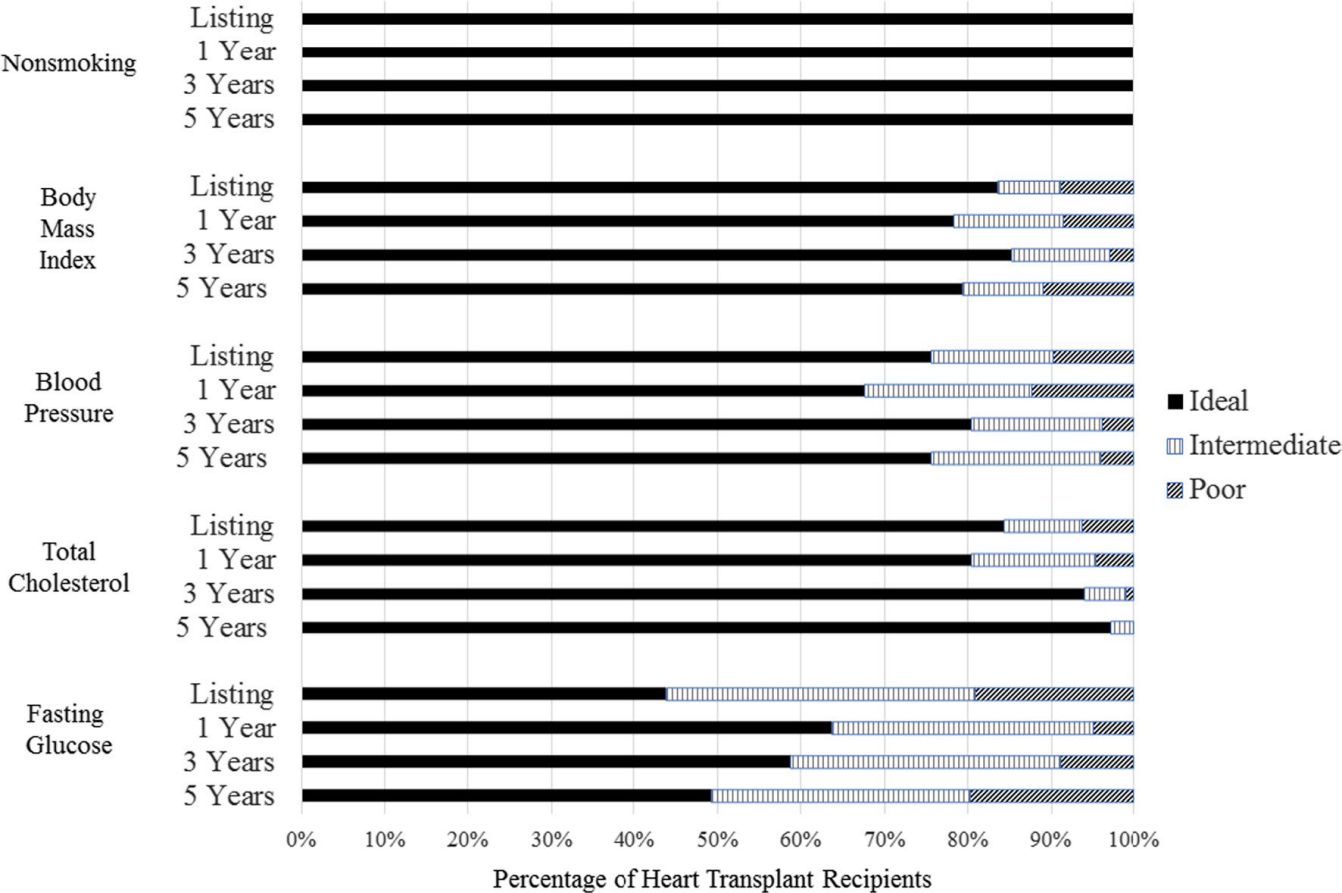
Cardiovascular Health Individual Metric Distributions in Heart Transplant Recipients, By Time Point

Figure 1 shows the distribution of individual CVH metric levels among HT recipients over time, and Additional file 1: eTable 1 provides additional details on mean metric levels and subgroup comparisons. The smoking metric was ideal (never smoked) among 100% of HT recipients at all points assessed. Most HT recipients had ideal levels of BMI (78–84%), BP (98–100%), and TC (79–95%). Conversely, only about one-half of HT recipients had ideal fasting blood glucose (44–64%), with 5–20% having poor glucose levels (the remainder were intermediate; Fig. 1, Additional file 1: eTable 1). Of the patients that were noted to have intermediate or poor BP, 48% were on blood pressure medication. There were 17% of patients with intermediate or poor TC or LDL-C that were placed on statin prophylactically. None of the CVH metric distributions changed significantly over time, from listing to 5-years post-HT.

**Fig. 2:**
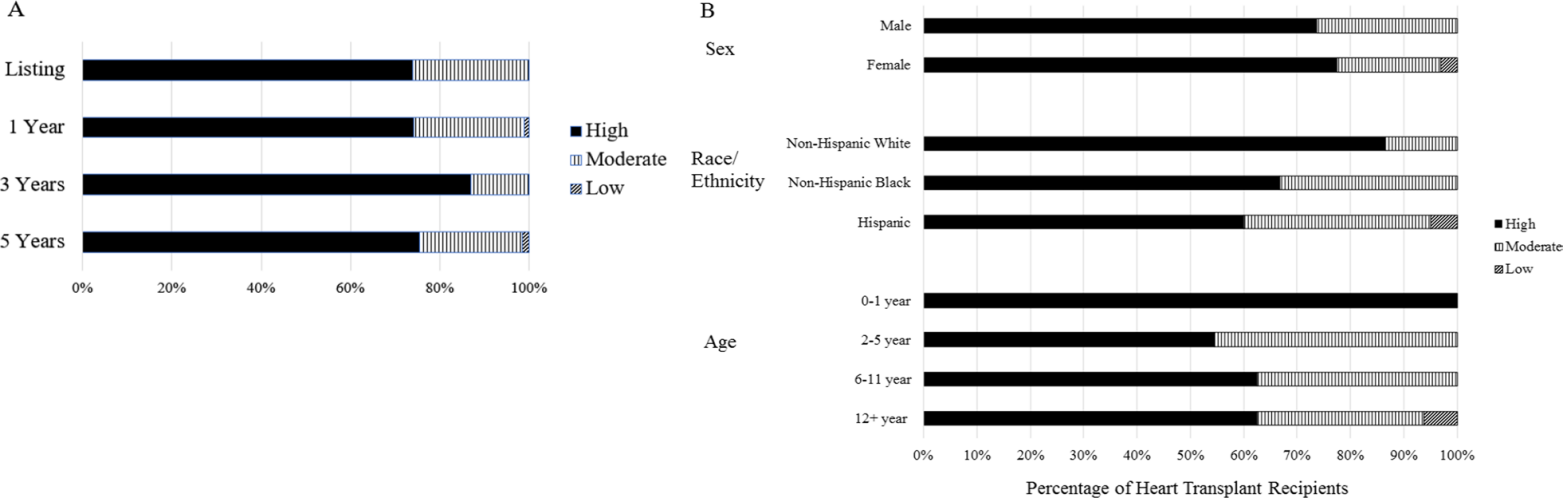
Composite Cardiovascular Health Score Distributions Among Heart Transplant Recipients, By Time Point and Subgroup. **A** Composite cardiovascular health score distributions among all heart transplant recipients, by time point. **B** Composite cardiovascular health score distributions among subgroups of heart transplant recipients at post-transplant Year 5

Figure 2A shows the distribution of composite CVH scores (high, moderate, low) among HT recipients over time, and Table 3 provides additional detail. As with the individual CVH metrics, the majority of HT recipients had high composite CVH score across all time points, with mean scores of 7.9–8.8 (out of 10) over time. There was no significant difference in composite CVH scores over time.

**Table 3:** Cardiovascular health score in heart transplant recipients, by time point and subgroup

	Composite cardiovascular health scores															
	At listing				1 year post-transplant				3 years post-transplant				5 years post-transplant			
	N	Mean (SD)	High, N (%)	Low, N (%)	N	Mean (SD)	High, N (%)	Low, N (%)	N	Mean (SD)	High, N (%)	Low, N (%)	N	Mean (SD)	High, N (%)	Low, N (%)
Overall	24	8.0 (1.0)	18 (75)	0 (0)	86	8.4 (1.5)	64 (74)	1 (1)	100	8.8 (1.3)	87 (87)	0 (0)	70	8.4 (1.5)	53 (76)	1 (1)
Males	14	8.0 (1.0)	10 (77)	0 (0)	51	8.4 (1.3)	39 (77)	0 (0)	56	8.8 (1.2)	50 (89)	0 (0)	38	8.4 (1.4)	28 (74)	0 (0)
Females	10	8.2 (1.6)	7 (7)	0 (0)	35	8.3 (1.7)	24 (71)	1 (3)	44	8.9 (1.4)	36 (84)	0 (0)	32	8.4 (1.6)	24 (77)	1 (3)
By age at transplant, years																
0–1	3	9.3 (0.6)	3 (100)	0 (0)	23	8.7 (1.3)	20 (87)	0 (0)	34	9.3 (1.0)	31 (91)	0 (0)	26	9.3 (0.8)	26 (100)	0 (0)
2–5	2	8.5 (0.7)	2 (100)	0 (0)	11	8.5 (1.6)	8 (73)	0 (0)	15	9.2 (0.9)	15 (100)	0 (0)	11	8.0 (1.1)	6 (55)	0 (0)
6–11	12	8.1 (1.6)	8 (67)	0 (0)	22	8.5 (1.3)	15 (71)	0 (0)	22	8.6 (1.4)	18 (86)	0 (0)	17	7.9 (1.4)	10 (63)	0 (0)
12+	7	8.0 (1.0)	4 (67)	0 (0)	30	8.0 (1.6)	20 (67)	1 (3)	29	8.3 (1.4)	22 (76)	0 (0)	16	7.8 (2.0)	10 (63)	1 (6)
By race/ethnicity																
NH White	12	8.6 (1.4)	9 (75)	0 (0)	47	8.7 (1.3)	38 (81)	0 (0)	57	8.5 (1.4)	53 (93)	0 (0)	37	8.7 (1.0)	32 (87)	0 (0)
NH Black	6	8.2 (1.8)	4 (67)	0 (0)	19	8.0 (1.3)	12 (63)	0 (0)	18	8.4 (1.5)	15 (83)	0 (0)	12	8.3 (1.8.3)	8 (67)	0 (0)
Hispanic	6	8.0 (1.0)	4 (80)	0 (0)	19	7.9 (1.9)	13 (68)	1 (5)	24	9.1 (1.1)	18 (75)	0 (0)	20	7.9 (2.0)	12 (60)	1 (5)
By cardiac diagnosis																
CM	11	8.0 (1.0)	8 (73)	0 (0)	50	8.4 (1.6)	37 (76)	1 (2)	58	8.8 (1.4)	48 (84)	0 (0)	42	8.5 (1.6)	32 (78)	1 (2)
CHD	13	8.4 (1.5)	10 (77)	0 (0)	32	8.3 (1.3)	23 (72)	0 (0)	38	8.9 (1.0)	35 (92)	0 (0)	26	8.2 (1.3)	18 (69)	0 (0)

*CHD* congenital heart disease, *CM* cardiomyopathy, *NH* non-Hispanic

*Smoking metric not included

In exploratory subgroup analyses, we explored relationships of patient age, race/ethnicity, sex and diagnosis with CVH and its trends over time post-HT. Younger patients (0–1 years) had higher prevalence of ideal BMI (92–100%) compared to older patients (12 + years: 55–67%; Additional file 2: eTable 2), and older patients (12 + years) also had a higher prevalence of a poor composite CVH score (2–6%) compared with younger patients (Fig. 2B). Non-Hispanic Black HT recipients had a lower prevalence of ideal BMI (58–78%) compared to the other race/ethnicity groups (67–92%; Additional file 2: eTable 2), but poor overall CVH scores were most prevalent among the Hispanic subgroup (up to 5%; Fig. 2B). CVH metric distributions and trends over time post-HT were not substantially different by sex or cardiac diagnosis.

### Comparison of CVH between HT Recipients and the General Population

**Fig. 3:**
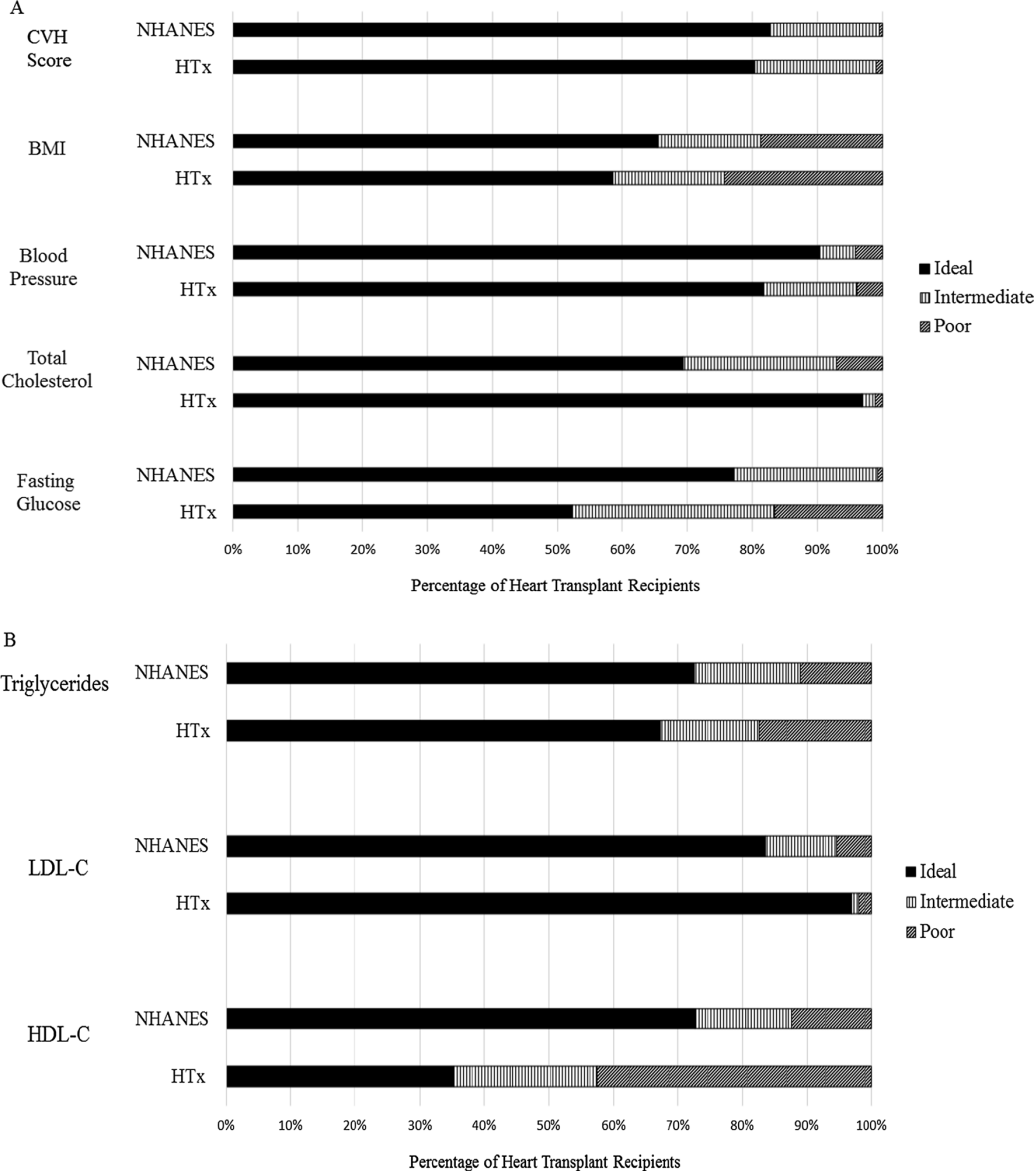
Cardiovascular Health Metrics and Lipid Fractions Among Heart Transplant Recipients (at Years 3–5) versus the General Population (NHANES). **A** Cardiovascular health metric distributions are shown among transplant recipients at the latest point of follow up (year 3 or 5 post-transplant), versus the age-, sex-, and race/ethnicity-adjusted general population of United States children, using weighted survey data from the National Health and Nutrition Examination Survey, 2007–2013. See text (Methods) for details. **B** Cardiovascular health lipid fractions among transplant recipients at the latest point of follow up (year 3 or 5 post-transplant), versus age-, sex- and race/ethnicity-adjusted general population of United States children, using weighted survey data from the National Health and Nutrition Examination Survey, 2007–2013. *CVH score* cardiovascular health score, *BMI* body mass index, *HDL-C* high density lipoprotein-calculated, *HTx* heart transplant recipients, *LDL-C* low density lipoprotein-calculated, *NHANES* National Health and Nutrition Examination Survey

Figure 3 and Additional file 2: eTable 2 show levels of CVH metrics, CVH scores, and lipid fractions among HT recipients and similarly-aged children in the GP. The distribution of composite CVH scores was generally similar between the two groups, as were the distributions of BMI and BP (Fig. 3A). Conversely, the distributions of TC and fasting glucose differed. HT recipients had a higher prevalence of ideal TC compared to the GP (97% vs 69%; Fig. 3A). Poor TC was present among only 1% of the HT group, versus 7% in the GP. Mean levels of TC were also higher in the GP (157 vs. 120 mg/dL; Additional file 2: eTable 2). In secondary analyses of lipid fractions, HT recipients had lower LDL-C (mean 65 vs. 87 mg/dL; Additional file 2: eTable 2), but also lower HDL-C (mean 42 vs. 53 mg/dL) when compared to the GP (Fig. 3B). Fasting blood glucose levels were less favorable among HT recipients than GP, with ideal levels present in 52% versus 77% and mean levels of 107 and 95 mg/dL, respectively (Fig. 3A, Additional file 2: eTable 2).

### Association of CVH with adverse outcomes among HT recipients

A total of 86 HT patients had full CVH score at 1-year post-transplant with 16 adverse outcomes (11 deaths, 4 CKD, 1 CAV) occurring between 1 and 3 years post-transplant that were included in analysis. We examined patients that were on BP medication and had a history of dyslipidemia prior to statin initiation and found no increased risk to CAV, CKD or death. Among HT patients with a full CVH score available at 1-year PT and outcomes ascertained at 3-years PT, there was no statistically significant association between year 1 CVH score and the odds for the composite adverse HT outcome in unadjusted (OR 0.87, 95% CI 0.6–1.3) or adjusted analysis (OR 0.95, 95% CI 0.7–1.4).

## Discussion

To our knowledge, this is the first study to examine CVH (as defined by the AHA) in the pediatric HT population. In this relatively small analysis of data from 110 HT patients, most HT patients had high (favorable) levels of individual CVH metrics and composite CVH scores with no significant declines over time through 5-years post-HT. In the sub-group analyses older children and minorities held lower CVH metrics compared to their counterparts. When compared to the US general population of similarly aged children, fasting glucose and HDL-C levels were worse among the HT group, whereas TC and LDL-C were better among the HT group. Among the subset of 86 HT patients with available data, we were unable to detect a significant association between CVH score at 1-year PT and the composite adverse HT outcome (re-listing, death, CAV or CKD) at 3-years PT.

Previous studies examining individual CVH metrics among pediatric HT recipients have been somewhat inconsistent, with some but not all suggesting worse levels of CVH metrics among HT recipients compared with the general population. Suboptimal nutrition and a sedentary lifestyle in combination with immunosuppressants and steroid medication are thought to be contributory to worse CVH metrics after HT [3, 8, 29, 31]. Several studies suggested increased risks of obesity, dyslipidemia and a higher incidence of type 2 diabetes post-HT [8, 13, 32–34]. For BMI, some studies have suggested increased obesity risks after HT, but a retrospective ISHLT registry study indicated that 8% of pediatric HT recipients were obese [34], whereas 17% of children were obese in the US general population based on NHANES data [17]. Our estimates of poor BMI (obesity) in HT recipients in the current study are very similar to findings from the ISHLT registry. For BP, limited data exist regarding the incidence of hypertension in pediatric HT recipients, although a single-center study found that 38% of pediatric HT recipients had a systolic blood pressure above the 95th percentile [35]. Our single-center study found much lower prevalence of poor BP (hypertension), and further study is needed. Lipids have been more extensively studied among HT recipients. Statin therapy was noted to help prevent the development of CAV [9] and thus initiation of statin therapy in children began to be more widespread in 2010 [9, 10, 12]. A PHTS study in 2004 (prior to widespread statin use for prevention of CAV) found that up to 43% of HT patients had TC > 200 mg/dL [8], which reflects the immunosuppressant changes over time, as cyclosporine and steroids have been noted to be independent risk factors for dyslipidemia [7] that are less commonly used while statin therapy has been effective in lowering TC and are more integrated in post-HT management in the current era [8]. A multi-institutional study in 2006 noted no significant difference in dyslipidemia between the US GP and the pediatric HT population [7], and registry studies have reported similar findings to our study in which the majority of HT recipients had a normal BMI and TC at time of transplant [7, 34]. Our more favorable findings for TC and LDL-C likely reflect standard use of statins. However, it is notable that HT recipients had poorer HDL-C levels compared with GP, and this could be due to differences in diet and physical activity levels, which we were not able to measure. For glucose, one study reported a high incidence of 11% of type 2 diabetes mellitus post-HT after 10 years of follow-up [36]. We found poorer glucose levels among HT recipients compared with GP, but in our time frame of follow-up we did not find a high prevalence of poor glucose (diabetes). An important factor to consider in the comparing studies is era and related changes in medication use. For example, studies have shown that when compared to cyclosporine, tacrolimus use was associated with a lower incidence of type 2 diabetes but a higher incidence of dyslipidemia [7, 36]. Our study’s findings demonstrate not only multiple CVH metrics for HT recipients, but also provide data that is reflective of the current HT immunosuppressant and statin use regimen of this era.

In this single-center study, we did not detect a significant association between CVH score at 1 year post-transplant and adverse outcomes over the following 2 years. Previous studies have shown the association of dyslipidemia with CAV [5, 7, 13, 31] as well as independent associations of diabetes and obesity with adverse outcomes in the pediatric HT population [8, 35, 36]. Given multiple previous studies have shown how various CVH metrics can contribute to adverse long-term outcomes in the pediatric HT population [1, 5, 8, 9, 12], our findings likely reflect lack of power. Future studies with a larger sample size and longer duration of follow-up are needed to further explore associations between suboptimal CVH metrics and adverse HT outcomes. Such studies could also determine whether particular subgroups (e.g. older patients and minorities) remain at significant risk.

### Limitations

There are inherent limitations to a single center retrospective study. First, our findings may not be generalizable to other centers and overall sample size was limited. A larger multicenter cohort would allow meaningful analyses among patient sub-groups, and is planned. Second, we were not able to analyze two of the seven CVH metrics, diet and physical activity, as this information was not consistently available in medical records. Prospectively capturing these two metrics may be useful. Third, statins were more widely used at our institution during the later period of the study, which could have affected the lipid profiles, we attempted to account for medication use in categorical analyses by subtracting a point from the corresponding CVH score, but we did not adjust continuous lipid levels for statin use. Fourth, the duration of the follow-up available for this study may not have been long enough to capture CAV or late mortality.

## Conclusion

We found that over the first 5-years post-HT, most HT recipients had high CVH scores. However, lipids and glucose were less optimal than the other metrics, and older patients and racial minorities appeared to have lower levels in some metrics. Given prior evidence for the importance of individual CVH metrics for HT outcomes, further study of CVH among pediatric HT patients is warranted.

## Supplementary Information

**Additional file 1: eTable 1.** Cardiovascular Health in Heart Transplant Recipients, by Time Point and Subgroup.

**Additional file 2: eTable 2.** Cardiovascular Health in Transplant Recipients Versus the General Population.

## Data Availability

The datasets generated during and analyzed during the current study are available from the corresponding author on reasonable request.
