# Supplementary material for: Cardiovascular health in pediatric heart transplant patients
Source: BMC Cardiovasc Disord. 2022 Apr 1;22:139. doi: 10.1186/s12872-022-02575-z (PMC8973961; doi:10.1186/s12872-022-02575-z)
Supplement: Supplementary file 1 — Additional file 1: eTable 1. Cardiovascular Health in Heart Transplant Recipients, by Time Point and Subgroup. [file 12872_2022_2575_MOESM1_ESM.docx]

eTable 1. Cardiovascular Health in Heart Transplant Recipients, by Time Point and Subgroup

|  | **Individual Cardiovascular Health Metrics** | | | | | | | | | | | | | | | |
| --- | --- | --- | --- | --- | --- | --- | --- | --- | --- | --- | --- | --- | --- | --- | --- | --- |
|  | **At Listing** | | | | **1 Year Post-Transplant** | | | | **3 Years Post-Transplant** | | | | **5 Years Post-Transplant** | | | |
|  | **N** | **Mean (SD)** | **Ideal,**  **N (%)** | **Poor,**  **N (%)** | **N** | **Mean (SD)** | **Ideal,**  **N (%)** | **Poor,**  **N (%)** | **N** | **Mean (SD)** | **Ideal,**  **N (%)** | **Poor,**  **N (%)** | **N** | **Mean (SD)** | **Ideal,**  **N (%)** | **Poor,**  **N (%)** |
| **Body Mass Index Percentile** | | | | | | | | | | | | | | | | |
| **Overall** | 87 | 17 (5) | 66 (84) | 7 (9) | 106 | 19 (5) | 83 (78) | 9 (9) | 102 | 19 (5) | 82 (80) | 4 (4) | 75 | 19 (6) | 58 (79) | 8 (11) |
| **Males** | 47 | 18 (5) | 34 (79) | 5 (12) | 60 | 19 (5) | 45 (75) | 7 (12) | 57 | 19 (5) | 47 (82) | 3 (5) | 40 | 19 (5) | 31 (79) | 5 (13) |
| **Females** | 40 | 16 (4) | 32 (89) | 2 (6) | 46 | 18 (6) | 38 (83) | 2 (4) | 45 | 18 (5.0) | 40 (89) | 0 (0) | 35 | 19 (6) | 27 (79) | 3 (9) |
| **By Age at Transplant, Years** | | | | | | | | | | | | | | | | |
| **0-1** | 32 | 15 (2) | 25 (96) | 1 (4) | 37 | 17 (3) | 34 (92) | 2 (5) | 36 | 16 (1) | 36 (100) | 0 (0) | 27 | 15 (1) | 27 (100) | 0 (0) |
| **2-5** | 12 | 16 (1) | 10 (91) | 0 (0) | 15 | 16 (2) | 14 (93) | 0 (0) | 15 | 16 (1) | 15 (100) | 0 (0) | 11 | 17 (3) | 9 (82) | 1 (9) |
| **6-11** | 21 | 18 (5) | 17 (81) | 2 (10) | 23 | 20 (7) | 18 (78) | 2 (9) | 22 | 20 (6) | 17 (77) | 1 (5) | 19 | 22 (7) | 10 (59) | 4 (24) |
| **12+** | 22 | 20 (6) | 14 (67) | 3 (14) | 31 | 23 (6) | 17 (55) | 4 (13) | 29 | 23 (5) | 19 (66) | 2 (7) | 18 | 23 (5) | 12 (67) | 3 (17) |
| **By Race/Ethnicity** | | | | | | | | | | | | | | | | |
| **NH White** | 51 | 16 (4) | 40 (91) | 3 (7) | 61 | 17 (4) | 54 (89) | 1 (2) | 59 | 18 (4) | 54 (92) | 1 (2) | 40 | 17 (4) | 36 (92) | 1 (3) |
| **NH Black** | 15 | 20 (6) | 10 (67) | 4 (27) | 20 | 22 (7) | 12 (60) | 6 (30) | 18 | 22 (6) | 14 (78) | 1 (6) | 12 | 22 (9) | 7 (58) | 4 (33) |
| **Hispanic** | 20 | 18 (5) | 15 (79) | 0 (0) | 24 | 20 (5) | 16 (67) | 2 (8) | 24 | 19 (5) | 18 (75) | 1 (4) | 22 | 20 (6) | 14 (67) | 3 (14) |
| **By Cardiac Diagnosis** | | | | | | | | | | | | | | | | |
| **CM** | 48 | 18 (6) | 35 (81) | 5 (12) | 61 | 20 (6) | 45 (74) | 6 (10) | 58 | 19 (6) | 49 (84) | 2 (3) | 47 | 20 (6) | 35 (78) | 6 (13) |
| **CHD** | 37 | 17 (3) | 30 (88) | 2 (6) | 41 | 18 (4) | 35 (85) | 2 (5) | 40 | 18 (4) | 35 (88) | 0 (0) | 26 | 19 (5) | 21 (81) | 2 (7) |
| **Systolic Blood Pressure (mmHg)** | | | | | | | | | | | | | | | | |
| **Overall** | 82 | 93 (14) | 81 (98) | 1 (1) | 106 | 104 (13) | 86 (98) | 2 (1) | 102 | 103 (12) | 100 (98) | 1 (1) | 74 | 105 (12) | 74 (100) | 0 (0) |
| **Males** | 43 | 97 (13) | 47 (96) | 1 (2) | 60 | 105 (13) | 58 (97) | 2 (3) | 57 | 105 (12) | 55 (96) | 1 (2) | 40 | 106 (13) | 40 (100) | 0 (0) |
| **Females** | 39 | 89 (13) | 39 (100) | 0 (0) | 46 | 102 (13) | 46 (100) | 0 (0) | 45 | 99 (9) | 45 (100) | 0 (0) | 34 | 104 (11) | 34 (100) | 0 (0) |
| **By Age at Transplant, Years** | | | | | | | | | | | | | | | | |
| **0-1** | 31 | 86(13) | 31 (100) | 0 (0) | 37 | 97 (12) | 36 (97) | 1 (3) | 36 | 97 (9) | 35 (97) | 1 (3) | 27 | 96 (9) | 27 (100) | 0 (0) |
| **2-5** | 13 | 93 (16) | 13 (100) | 0 (0) | 15 | 96 (9) | 13 (93) | 1 (7) | 15 | 99 (10) | 15 (100) | 0 (0) | 11 | 107 (6) | 11(100) | 0 (0) |
| **6-11** | 18 | 95 (8) | 19 (95) | 0 (0) | 23 | 106 (8) | 23 (100) | 0 (0) | 22 | 103(8) | 22 (100) | 0 (0) | 19 | 110 (13) | 19 (100) | 0 (0) |
| **12+** | 20 | 103(14) | 23 (96) | 1 (4) | 31 | 114 (10) | 31 (100) | 0 (0) | 29 | 112(11) | 28 (97) | 0 (0) | 17 | 112 (12) | 17 (100) | 0 (0) |
| **By Race/Ethnicity** | | | | | | | | | | | | | | | | |
| **NH White** | 49 | 92 (15) | 53 (98) | 1 (2) | 61 | 102 (12) | 59 (97) | 2 (3) | 59 | 101 (11) | 57 (97) | 1 (2) | 39 | 105 (11) | 39 (100) | 0 (0) |
| **NH Black** | 13 | 97 (12) | 14 (100) | 0 (0) | 20 | 107 (12) | 20 (100) | 0 (0) | 18 | 105 (14) | 18 (100) | 0 (0) | 12 | 101 (14) | 12 (100) | 0 (0) |
| **Hispanic** | 19 | 95 (15) | 18 (95) | 0 (0) | 24 | 107 (14) | 24 (100) | 0 (0) | 24 | 105 (11) | 24 (100) | 0 (0) | 22 | 107 (13) | 22 (100) | 0 (0) |
| **By Cardiac Diagnosis** | | | | | | | | | | | | | | | | |
| **CM** | 45 | 90 (16) | 43 (96) | 1 (2) | 61 | 104 (14) | 59 (97) | 2 (3) | 58 | 103 (13) | 57 (98) | 1 (2) | 46 | 105 (13) | 46 (100) | 0 (0) |
| **CHD** | 35 | 97 (11) | 37 (100) | 0 (0) | 41 | 103 (11) | 41 (100) | 0 (0) | 40 | 102 (9) | 40 (100) | 0 (0) | 26 | 106 (11) | 26 (100) | 0 (0) |
| **Diastolic Blood Pressure (mmHg)** | | | | | | | | | | | | | | | | |
| **Overall** | 88 | 52 (18) | 86 (98) | 1 (1) | 105 | 63 (12) | 103 (98) | 1(1) | 101 | 64 (11) | 100 (99) | 1 (1) | 74 | 66 (9) | 74 (100) | 0 (0) |
| **Males** | 49 | 52 (21) | 47 (96) | 2 (2) | 59 | 63(14) | 57 (96) | 2 (3) | 56 | 65 (10) | 54 (96) | 1 (2) | 40 | 66 (9) | 40 (100) | 0 (0) |
| **Females** | 39 | 53(2) | 39 (100) | 0 (0) | 46 | 63 (10) | 46 (100) | 0 (0) | 45 | 63 (11) | 45 (100) | 0 (0) | 34 | 66 (10) | 34 (100) | 0 (0) |
| **By Age at Transplant, Years** | | | | | | | | | | | | | | | | |
| **0-1** | 31 | 51 (12) | 31 (100) | 0 (0) | 37 | 58 (16) | 36 (97) | 1 (3) | 35 | 61 (9) | 35 (97) | 1 (3) | 27 | 60 (10) | 27 (100) | 0 (0) |
| **2-5** | 13 | 55 (14) | 13 (100) | 0 (0) | 14 | 61(6) | 14 (93) | 1 (7) | 15 | 62 (10) | 15 (100) | 0 (0) | 11 | 70 (5) | 11 (100) | 0 (0) |
| **Diastolic Blood Pressure (mmHg), by Age at Transplant in Years** | | | | | | | | | | | | | | | | |
|  | At Listing | | | | 1 Year Post-Transplant | | | | 3 Years Post-Transplant | | | | 5 Years Post-Transplant | | | |
|  | **N** | **Mean,**  **(SD)** | **Ideal,**  **N (%)** | **Poor,**  **N(%)** | **N** | **Mean,**  **(SD)** | **Ideal,**  **N (%)** | **Poor,**  **N(%)** | **N** | **Mean,**  **(SD)** | **Ideal,**  **N (%)** | **Poor,**  **N(%)** | **N** | **Mean,**  **(SD)** | **Ideal,**  **N (%)** | **Poor,**  **N(%)** |
| **6-11** | 20 | 52 (19) | 19 (95) | 0 (0) | 23 | 66 (7) | 23 (100) | 0 (0) | 22 | 63 (11) | 22 (100) | 0 (0) | 19 | 67(8) | 19 (100) | 0 (0) |
| **12+** | 24 | 53 (25) | 23 (96) | 1 (4) | 31 | 68 (9) | 31 (100) | 0 (0) | 29 | 70 (10) | 28 (97) | 1 (3) | 17 | 71(8) | 17 (100) | 0 (0) |
| **By Race/Ethnicity** | | | | | | | | | | | | | | | | |
| **NH White** | 54 | 49 (18) | 53 (98) | 1 (2) | 61 | 62 (14) | 59 (97) | 2 (3) | 59 | 64 (10) | 57 (97) | 1 (2) | 39 | 66 (9) | 39 (100) | 0 (0) |
| **NH Black** | 13 | 54 (15) | 13 (100) | 0 (0) | 20 | 63 (10) | 20 (100) | 0 (0) | 18 | 63 (13) | 18 (100) | 0 (0) | 12 | 63 (12) | 12 (100) | 0 (0) |
| **Hispanic** | 19 | 61 (16) | 18 (95) | 0 (0) | 24 | 65 (11) | 24 (100) | 0 (0) | 24 | 66 (10) | 24 (100) | 0 (0) | 22 | 67 (9) | 22 (100) | 0 (0) |
| **By Cardiac Diagnosis** | | | | | | | | | | | | | | | | |
| **CM** | 48 | 52 (19) | 46 (96) | 1 (2) | 60 | 64 (12) | 59 (98) | 1 (2) | 57 | 63 (11) | 56 (98) | 1 (2) | 46 | 66 (9) | 46 (100) | 0 (0) |
| **CHD** | 37 | 54 (150) | 37 (100) | 0 (0) | 41 | 61 (13) | 41 (100) | 0 (0) | 40 | 65 (8) | 40 (100) | 0 (0) | 26 | 65 (10) | 26 (100) | 0 (0) |
| **Total Cholesterol (mg/dL)** | | | | | | | | | | | | | | | | |
| **Overall** | 31 | 131 (39) | 39 (89) | 2 (5) | 82 | 142 (41) | 65 (79) | 4 (5) | 66 | 124 (30) | 60 (91) | 1 (2) | 37 | 119 (27) | 35 (95) | 0 (0) |
| **Males** | 18 | 132 (39) | 16 (84) | 1 (5) | 50 | 141(38) | 42 (81) | 2 (4) | 51 | 122 (25) | 54 (96) | 0 (0) | 36 | 122 (26) | 38 (97) | 0 (0) |
| **Females** | 13 | 129 (42) | 11 (85) | 1 (8) | 35 | 144 (46) | 28 (80) | 2 (6) | 45 | 126 (34) | 41 (91) | 1 (2) | 32 | 115 (29) | 32 (97) | 0 (0) |
| **By Age at Transplant, Years** | | | | | | | | | | | | | | | | |
| **0-1** | 3 | 121 (20) | 3 (100) | 0 (0) | 22 | 134 (52) | 20 (87) | 2 (9) | 34 | 119 (33) | 33 (94) | 1 (3) | 25 | 113 (26) | 26 (100) | 0 (0) |
| **2-5** | 5 | 126 (37) | 4 (80) | 0 (0) | 11 | 137 (24) | 10 (91) | 0 (0) | 15 | 136 (25) | 13 (87) | 0 (0) | 11 | 127 (18) | 11 (100) | 0 (0) |
| **6-11** | 12 | 122 (31) | 12 (100) | 0 (0) | 23 | 146(31) | 17 (74) | 1 (4) | 21 | 119 (24) | 21 (95) | 0 (0) | 17 | 123 (34) | 17 (100) | 0 (0) |
| **12+** | 11 | 146 (50) | 8 (73) | 2 (18) | 29 | 148 (45) | 23 (79) | 4 (14) | 26 | 127 (30) | 19 (73) | 2 (8) | 15 | 116 (27) | 12 (80) | 3 (20) |
| **By Race/Ethnicity** | | | | | | | | | | | | | | | | |
| **NH White** | 15 | 137 (38) | 13 (81) | 1 (6) | 46 | 140 (39) | 40 (85) | 2 (4) | 56 | 124 (31) | 54 (93) | 1 (2) | 37 | 112 (25) | 37 (100) | 0 (0) |
| **NH Black** | 7 | 123 (31) | 7 (100) | 0 (0) | 18 | 140 (34) | 13 (74) | 1 (5) | 18 | 121 (24) | 18 (100) | 0 (0) | 19 | 134 (33) | 11 (92) | 0 (0) |
| **Hispanic** | 9 | 126 (49) | 7 (78) | 1 (11) | 20 | 146 (52) | 16 (80) | 1 (5) | 22 | 125 (29) | 23 (96) | 0 (0) | 21 | 122 (25) | 20 (95) | 0 (0) |
| **By Cardiac Diagnosis** | | | | | | | | | | | | | | | | |
| **CM** | 41 | 117 (28) | 12 (86) | 0 (0) | 54 | 123 (27) | 40 (78) | 3 (6) | 50 | 144 (43) | 56 (97) | 0 (0) | 13 | 126 (31) | 43 (98) | 0 (0) |
| **CHD** | 17 | 132 (45) | 15 (88) | 2 (12) | 31 | 137 (41) | 28 (88) | 1 (3) | 38 | 124 (33) | 35 (90) | 1 (3) | 25 | 119 (26) | 24 (96) | 1 (4) |
| **Fasting Blood Glucose (mg/dL)** | | | | | | | | | | | | | | | | |
| **Overall** | 89 | 109 (36) | 39 (44) | 17 (19) | 102 | 97 (15) | 65 (64) | 5 (5) | 102 | 101 (28) | 60 (59) | 9 (9) | 71 | 108 (34) | 35 (49) | 14 (20) |
| **Males** | 51 | 111 (26) | 18 (35) | 11 (22) | 57 | 98 (16) | 35 (61) | 4 (7) | 57 | 103 (28) | 30 (53) | 4 (7) | 38 | 109 (33) | 19 (50) | 8 (21) |
| **Females** | 38 | 107 (46) | 21 (55) | 6 (16) | 45 | 96 (14) | 30 (67) | 1 (2) | 45 | 99 (28) | 30 (67) | 5 (11) | 33 | 108 (35) | 16 (48) | 6 (18) |
| **By Age at Transplant, Years** | | | | | | | | | | | | | | | | |
| **0-1** | 30 | 101 (18) | 13 (43) | 2 (7) | 34 | 94 (13) | 24 (71) | 0 (0) | 36 | 91 (27) | 27 (75) | 2 (6) | 27 | 98 (21) | 16 (59) | 4 (15) |
| **2-5** | 12 | 103 (21) | 5 (42) | 2 (17) | 15 | 98 (20) | 10 (67) | 2 (13) | 15 | 99 (12) | 7 (47) | 0 (0) | 11 | 115 (23) | 2 (18) | 3 (27) |
| **6-11** | 21 | 123 (59) | 9 (43) | 8 (38) | 23 | 98 (14) | 16 (70) | 1 (4) | 22 | 99 (17) | 12 (55) | 2 (9) | 17 | 105 (27) | 10 (59) | 3 (20) |
| **12+** | 26 | 111 (30) | 12 (46) | 5 (19) | 30 | 100 (16) | 15 (50) | 2 (7) | 29 | 116 (43) | 14 (48) | 5 (17) | 16 | 125 (54) | 7 (44) | 4 (25) |
| **By Race/Ethnicity** | | | | | | | | | | | | | | | | |
| **NH White** | 50 | 111 (44) | 23 (46) | 10 (20) | 59 | 97 (15) | 38(64) | 3 (5) | 59 | 98 (16) | 34 (58) | 4 (7) | 38 | 107 (32) | 18 (47) | 7 (18) |
| **NH Black** | 19 | 105 (21) | 8 (44) | 3 (17) | 20 | 97 (12) | 14 (70) | 0 (0) | 18 | 94 (16) | 13 (72) | 1 (6) | 12 | 103 (30) | 8 (67) | 2 (17) |
| **Hispanic** | 20 | 110 (23) | 7 (35) | 4 (20) | 22 | 100 (16) | 12 (55) | 2 (9) | 24 | 116 (49) | 12 (50) | 4 (17) | 20 | 116 (41) | 9 (45) | 5 (25) |
| **By Cardiac Diagnosis** | | | | | | | | | | | | | | | | |
| **CM** | 50 | 108 (42) | 25 (50) | 7 (14) | 58 | 96 (14) | 41 (71) | 2 (3) | 58 | 101 (31) | 38 (66) | 4 (7) | 43 | 103 (28) | 24 (56) | 5 (12) |
| **CHD** | 35 | 113 (25) | 12 (34) | 10 (29) | 40 | 100 (17) | 20 (50) | 3 (8) | 40 | 103 (24) | 19 (48) | 5 (13) | 26 | 120 (41) | 9 (35) | 9 (35) |

CHD, congenital heart disease; CM, cardiomyopathy; NH, non-Hispanic.

*Smoking metric not included
