# Supplementary material for: Cardiovascular health in pediatric heart transplant patients
Source: BMC Cardiovasc Disord. 2022 Apr 1;22:139. doi: 10.1186/s12872-022-02575-z (PMC8973961; doi:10.1186/s12872-022-02575-z)
Supplement: Supplementary file 2 — Additional file 2: eTable 2. Cardiovascular Health in Transplant Recipients Versus the General Population. [file 12872_2022_2575_MOESM2_ESM.docx]

eTable 2. Cardiovascular Health in Transplant Recipients Versus the General Population

|  | **Cardiovascular Health Metric Level, Mean** | | | | | | | | | | | | | | | |
| --- | --- | --- | --- | --- | --- | --- | --- | --- | --- | --- | --- | --- | --- | --- | --- | --- |
|  | **BMI %ile** | | **SBP %ile** | | **DBP %ile** | | **TC (mg/dL)** | | **LDL-C (mg/dL)** | | **TG (mg/dL)** | | **HDL-C (mg/dL)** | | **FBG**  **(mg/dL)** | |
|  | **HTx (SD)** | **GP**  **(SE)** | **HTx (SD)** | **GP (SE)** | **HTx (SD)** | **GP (SE)** | **HTx (SD)** | **GP (SE)** | **HTx (SD)** | **GP (SE)** | **HTx (SD)** | **GP (SE)** | **HTx (SD)** | **GP (SE)** | **HTx (SD)** | **GP (SE)** |
| **Overall** | 66 (5.6) | 64 (0.4) | 55 (31) | 49 (0.6) | 68 (29) | 35 (0.8) | 120 (29) | 157 (0.5) | 65 (41) | 87 (0.7) | 83 (57) | 80 (1.8) | 42 (15) | 53 (0.2) | 107 (35) | 95 (0.5) |
| **Males** | 67 (32) | 63 (0.5) | 54 (31) | 50 (0.8) | 71 (27) | 33 (0.8) | 121 (25) | 156 (0.5) | 64 (20) | 85 (1.2) | 85.4 (39) | 81 (2.6) | 41 (16) | 53 (0.3) | 109 (36) | 97 (0.9) |
| **Females** | 65 (33) | 64 (0.5) | 55 (31) | 48 (0.7) | 64 (33) | 37 (1) | 118 (33) | 159 (0.6) | 67 (57) | 88.7 (0.9) | 80.7 (71) | 79 (2.3) | 43 (14) | 53 (0.3) | 103 (32) | 93 (0.4) |
| **By age at transplant, years** | | | | | | | | | | | | | | | | |
| **0-1** | 17 (21) | 76 (0.7) | 53 (31) |  | 83 (22) |  | 116 (32) |  | 62 (26) |  | 60 (28) |  | 41 (11) |  | 96 (20) |  |
| **2-5** | 61 (29) | 60 (0.6) | 65 (23) |  | 66 (26) |  | 126 (17) |  | 68 (12) |  | 65 (20) |  | 46 (18) |  | 108 (23) |  |
| **6-11*** | 69 (31) | 63 (0.6) | 59 (30) | 53 (0.8) | 75 (19) | 32 (1) | 120 (31) | 160 (0.6) | 55 (22) |  | 103 (51) |  | 45 (21) | 55 (0.3) | 103 (25) |  |
| **12+** | 69 (33) | 66 (0.6) | 50 (36) | 46 (0.7) | 54 (34) | 36 (0.9) | 121 (30) | 155 (0.6) | 74 (69) | 87 (0.7) | 108 (80) | 80 (1.8) | 38 (11) | 52 (0.3) | 121 (51) | 95 (0.5) |
| **By race/ethnicity** | | | | | | | | | | | | | | | | |
| **NH white** | 59 (33) | 62 (0.5) | 57 (30) | 47 (0.8) | 65 (30) | 35 (1.2) | 116 (30) | 157 (0.8) | 61 (23) | 87 (1.2) | 77 (39) | 82 (2.8) | 40 (18) | 52 (0.4) | 103 (28) | 95 (0.8) |
| **NH black** | 81 (23) | 67 (0.7) | 47 (31) | 52 (0.8) | 79 (24) | 35 (1.1) | 129 (30) | 157 (0.7) | 67 (25) | 87 (1.1) | 69 (31) | 61 (1.7) | 49 (8) | 57 (0.4) | 103 (26) | 93 (0.6) |
| **Hispanic** | 69 (33) | 69 (0.6) | 57 (35) | 51 (1) | 76 (32) | 35 (0.9) | 120 (25) | 156 (0.8) | 74 (74) | 86 (1.7) | 105 (87) | 84 (2.5) | 41 (9) | 52 (0.4) | 120 (51) | 97 (0.9) |

Cardiovascular health metric distributions are shown among transplant recipients at the latest point of follow up (year 3 or 5 post-transplant), versus age-, sex-, and race/ethnicity-adjusted general population of United States children, using weighted survey data from the National Health and Nutrition Examination Survey, 2007-2013. See text (Methods) for details.

BMI, body mass index; DBP, diastolic blood pressure; FBG, fasting blood glucose; GP, general population; HDL-C, high density lipoprotein-calculated; HTx, heart transplant recipients; LDL-C, low density lipoprotein-calculated; SBP, systolic blood pressure; TC, total cholesterol; TG, triglycerides; %, percentile

*SBP and DBP used for patients 8 years and older in the general population group
